# Evidence of Aujeszky’s disease in wild boar in Serbia

**DOI:** 10.1186/s12917-016-0758-9

**Published:** 2016-06-30

**Authors:** V. Milicevic, S. Radojicic, M. Valcic, V. Ivovic, V. Radosavljevic

**Affiliations:** Virology department, Institute of Veterinary Medicine of Serbia, Vojvode Toze 14, 11000 Belgrade, Serbia; Infectious Animals Diseases and Diseases of Bees, Faculty of Veterinary Medicine, Bulevar oslobodjenja 18, 11000 Belgrade, Serbia; Department of Biodiversity, University of Primorska, Faculty of Mathematics, Natural Sciences and Information Technologies, Glagoljaska 8, SI-6000 Koper, Slovenia; Institute of Veterinary Medicine of Serbia, Vojvode Toze 14, 11000 Belgrade, Serbia

**Keywords:** Aujeszky’s disease virus, PCR, Virus isolation, Virus neutralization test, Wild boar

## Abstract

**Background:**

Aujeszky’s disease is a viral disease of suids caused by *Suid Herpesvirus 1*. The disease has worldwide distribution with significant economic impact. In Serbia, there is neither an Aujeszky’s disease eradication nor national vaccination programme of domestic pigs.

Since clinical symptoms of Aujeszky’s disease are not specific, it is important to establish a link between clinical signs and presence of ADV active infection in wild boars. The aim of this study was to investigate the possibility of active infection within wild boar showing signs of ADV and also to examine relationship between isolates from domestic pigs and wild boar. Having in mind that virus has not been previously isolated from wild boars in Serbia, we report the first isolation of *Suid Herpesvirus 1* from this species in Serbia.

**Results:**

Tissue and serum samples from 40 wild boars from eastern Serbia were examined for evidence of Aujeszky’s disease (AD). *Suid Herpesvirus 1* (SHV1), the cause of AD was isolated on PK15 cell line from three tissue samples, inducing cytopathic effect (CPE) with syncytia forming, and viral genome was detected by polymerase chain reaction (PCR) in eight samples. Genetic analysis of us4, us9 and ul49.5 partial sequences showed high homology between ADV isolates from wild boars and between isolates from wild boars and domestic animals. Neutralizing antibodies were not detected by virus neutralisation test (VNT) in sera from four out of eight PCR positive wild boars suggesting recent infection in those animals.

**Conclusions:**

This is the first demonstration of Aujeszky’s disease virus (ADV) in the wild boar population in Serbia although seroconversion has been detected previously.

## Background

Aujeszky’s disease (AD) is a viral disease of suids caused by *Suid Herpesvirus 1* (SHV1) [1], also referred to as Aujeszky’s disease virus (ADV). The virus belongs to the genus Varicellovirus, subfamily Alphaherpesvirinae, family Herpesviridae. It has a double-stranded DNA genome composed of 143461 nucleotides with more than 70 open reading frames homologues to related Alphaherpesviruses [2]. Based on restriction fragment length polymorphism (RFLP) analysis patterns, ADV can be divided into four major genotypes [3]. But according to partial gC (ul44) coding region, it is possible to divide ADV into five genotypes that appear to be unspecific to countries or continents [4].

AD is also named pseudorabies (PR) and the virus Pseudorabies Virus (PRV), because carnivores and pigs may display neurological signs which can be similar to rabies.

Wild boar (*Sus scrofa*) are the natural host, but a wide range of species can be infected with SHV1 [5]. Wild boars are known as reservoirs for many important infectious diseases in domestic animals, such as classical swine fever, brucellosis and trichinellosis. Also, they can play a role of reservoirs for zoonotic diseases such as hepatitis E, tuberculosis, leptospirosis and trichinellosis [6]. Worldwide distribution, great potential of adaptation, fast reproductive rate and complex social behaviour make wild boar almost ideal reservoir species [7].

However, some diseases, like AD can sporadically occur in free living wild boars under natural conditions. Occurrence of these diseases is facilitated by social stress, age related change from passive to active immunity, individual susceptibility to ADV infection and environmental conditions [8].

AD has worldwide distribution [9]. The economic impact of AD is significant, consequently many developed countries (Belgium, Czech Republic, Denmark, Germany, Ireland, Cyprus, Luxembourg, Netherlands, Austria, Slovenia, Slovakia, Finland, Sweden, UK) have eradicated, or are in the process of eradication of the infection from domestic pigs^1^. AD may also be controlled nationally by a vaccination programme. In Serbia, there is neither an AD eradication nor national vaccination programme of domestic pigs. Vaccination is conducted on individual voluntary basis only. Only in commercial farms, pigs are regularly vaccinated to reduce potential losses due to AD and therefore AD has rarely been reported in swine.

In Serbia, intensive pig production is located in the north part of the country, in the Autonomous Province of Vojvodina. In this area AD has moderate impact on intensive pig production, with the 32.8 % ADV seroprevalence in unvaccinated breeding pigs [10]. Pusic et al. [10] demonstrated that the swine population in Vojvodina region, the most developed region in Serbia with the biosecurity measures most applied, was enzootically infected with ADV and that vaccination was only performed on large commercial farms. Those farms were usually surrounded by small backyard holdings with occasionally vaccinated animals, which presented a potential source of infection. Due to vaccination with attenuated vaccine of Bartha strain, which is not a marker vaccine, seroprevalence attributed to the natural infection is difficult to estimate. The sampling has been done in this region where the virus can be transferred from wild to domestic pigs since it is separated from Vojvodina only by the river Danube.

Clinical AD in wild boar has rarely been seen, although Gortazar et al. [11] described an outbreak in wild boar in Spain. It has been demonstrated that the virus could be successfully isolated from latently infected wild boars [12]. Serosurveillance studies have demonstrated that the prevalence of AD could be high within the European wild boar population, indicating a potentially significant wildlife reservoir of ADV [13]. Seroprevalence ranges from 0 % in the Netherlands and Sweden [14, 15] to more than 50 % in Croatia [16] and central Italy [17] and 100 % in Spain, on local level [18]. As documented in Germany, continuous parallel increase of both AD seroprevalence and wild boar population density implies the correlation of these two parameters [19].

Within the Classical Swine Fever (CSF) monitoring programme in Serbia, 20 % of wild boars tested annually for CSF were also tested for ADV antibodies. The Monitoring is issued annually by The Ministry of Agriculture and Environmental protection and prescribes the number of samples to be collected for each district. Hunting season in Serbia lasts from April 15th to February 28th for boars and young wild boars up to 60 kg and from July 1st to December 31st for sows, although hunting is the most intensive from November to February. Specified percent of wild boars were subjected to AD serology tests, either VNT or ELISA. ADV seroprevalence in different regions varies with the highest percentage found in the east - 83 % as average for the last three years (unpublished data).

Until 2011, vaccination of wild boar against classical swine fever has been performed in hunting grounds in Serbia. Wild boars have been trapped, vaccinated, ear tagged and released back in the nature. Classical swine fever vaccine used for wild boar vaccination was produced by local company and composed of CSF C strain and attenuated ADV Bartha strain viruses. Therefore, unintentionally, some wild boars had been vaccinated against AD. The wild boar population in Serbia is estimated at around 20000 animals, with density of 0.2 - 1.38 animals/km^2^.

Natural transmission of *Suid Herpesvirus 1* requires close contact between animals such as during coitus, licking, or nuzzling. Although, in high density commercial farms, sneezing and short distance droplet spreads are major routes of transmission [20].

The clinical presentation of ADV infection depends on the virulence and initial dose of virus; and also the age, immunological and reproductive status of the host [21]. Within wild boar, Gortazar et al. [11] reported that young animals were mostly affected, between four and eight months of age, with 14 % mortality. Although there is no evidence of any different susceptibility and disease course between wild and domestic pigs, it has been shown that strains from free-living wild boars differed genetically from those isolated from domestic pigs, but that both might have had a common origin [1, 22].

An important mechanism of ADV persistence, characteristic of all Alphaherpesviruses, is lifelong latency within the peripheral nervous system.

Diagnosis of Aujeszky’s disease can be achieved using various tests including viral isolation, molecular biology (PCR) and serology.

Since clinical symptoms of Aujeszky’s disease are not specific, it is important to establish a link between clinical signs and presence of ADV active infection in wild boars. The aim of this study was to investigate the possibility of active infection within wild boar showing signs of ADV and also to examine relationship between isolates from domestic pigs and wild boar. Having in mind that virus has not been previously isolated from wild boars in Serbia, we report the first isolation of *Suid Herpesvirus 1* from this species in Serbia.

## Methods

### Samples

Fourty samples were received from Eastern Serbia (44°22′1.417″N, 21°44′28.524″E) during the period November 2014 – January 2015, collected by hunters, for classical swine fever monitoring which was compulsory for each hunting ground. During that period, hunts were organised by local hunting associations every Sunday, and samples from each hunt were independently sent to the laboratory. According to the CSF monitoring plan, each sample set consisted of spleen, kidney, blood and, if possible, tonsils. Commonly used practice was that hunters at spot took samples and therefore, due to difficult sampling, tonsils were rarely submitted. Hunters were requested to fill in the form for each shot wild boar, specifying age, gender, previous vaccinations and a reason for hunt (injured, ill, sport). The hunters reported signs of illness as the reason for the kill of 18 wild boars, in independent hunts during the mentioned period. That high frequency of reported illness led to examinations for other viral diseases after CSF was excluded. Bearing in mind high AD seroprevalence in that region, samples were initially screened for ADV, first by PCR and then by virus isolation on PCR positive samples. Serum samples were tested for ADV antibodies by VNT.

The samples were also tested for *Porcine Circovirus 2* (PCV2), *Porcine Parvovirus* (PPV), *Swine Influenza Virus* (SIV), *Porcine Respiratory Corona virus* (PRCV) and *Porcine Reproductive and Respiratory Syndrome Virus* (PRRSV) by serological and molecular methods. For antibody detection, commercial ELISA kits were used for PCV2 (Ingezim Circovirus IgG/IgM, ING1.1.PCV.K.2, Ingenasa, Spain), SIV (Ingezim Influenza A, ING 1.0.FKU.K.3, Ingenasa, Spain), PRCV (Ingezim Corona Diferencial 2.0, 11.DIF.K3, Ingenasa, Spain) and PRRS (Ingezim PRRS Universal, ING 1.1PRU.K1, Ingenasa, Spain). Hemagglutination inhibition test was used for PPV antibody detection using reference strain NADL-2 (ATCC® VR-742) at 4HU. Genome detection of those viruses was performed following previously published protocols [23–25].

### DNA extraction and ADV PCR

DNA was extracted from pools of spleen and kidney tissues respectively using QIAamp DNA Mini Kit (Qiagen; Hilden, Germany), following the recommended tissue protocol.

The ul4 gene was amplified using previously published primers [26]. The PCR reaction was carried out in a total volume of 50 μL using HotStarTaq Master Mix Kit (Qiagen; Hilden, Germany) and 0.4 μM concentration of primers. The thermal profile for the PCR was 95 °C for 15 min, followed by 35 cycles of 95 °C for 1 min, 60 °C for 45 s and 72 °C for 1 min. The final extension step was performed at 72 °C for 5 min.

The amplified products were analysed by electrophoresis through 1.5 % agarose gel containing 0.5 mg/mL ethidium bromide. Each PCR run included positive and negative controls.

### DNA sequencing and sequence analysis

Eight PCR positive tissue samples from wild boars and three ADV isolates from domestic pigs were amplified with primers for us4, used in screening PCR, us9 and ul49.5 following a previously published protocol [27]. Amplified products were purified with MinElute PCR Purification Kit (Qiagen; Hilden, Germany) and sequenced by Macrogen Europe (Amsterdam, The Netherlands). For phylogenetic analysis, MEGA software version 6, Basic Local Alignment Searching Tool (BLAST) (http://blast.ncbi.nlm.nih.gov/Blast.cgi) and ADV sequences available in GeneBank (NCBI) were used.

Newly described ADV us4, us9 and ul49.5 sequences are available from GenBank under accession numbers KT187309-KT187326, KT273927-KT273941.

Within this study, three ADV strains isolated during the last 6 years from domestic animals - domestic pig/2009 (KT187312, KT187318, KT187324), dog/2010 (KT187313, KT187319, KT187325), domestic pig/2014 (KT187314, KT187320, KT187326) were submitted to the GenBank and used for genetic comparison with isolates from wild boars.

### Virus isolation and neutralisation

Positive PCR samples were prepared for virus isolation following the method described in OIE Terrestrial Manual (2012). Briefly, spleen and renal tissues were homogenized together in minimal essential medium with Earle’s salts and L glutamine (MEM, Gibco BRL, USA) with addition of antibiotics. Homogenates were centrifuged at 900 g for 10 minutes and supernatants were used for inoculation in 24 well plates (Sarstedt, Germany) with 80-100 % confluent monolayer of PK15 (BS CL 72, IZS, Brescia, Italy) cells maintained with 5 % newborn bovine serum (Gibco BRL, USA) and 5 % CO_2_ in air.

The cells were incubated at 37 °C and observed daily for CPE. In the samples where no CPE was observed, blind passage was performed by transferring of supernatant and inoculated tissue culture cells which were not demonstrating CPE to another plate containing fresh cells; in order to dilute out possible inhibitors and/or allow possible early viral replication due to low concentration of virus particles to progress to detectable CPE. If no CPE was evident after the second passage, the sample was considered negative.

To confirm the identity of the isolated virus, the cell culture supernatants were used in the neutralisation test with PRV S1 antiserum (CVI, Wageningen, Lelystad, Netherlands). The first aliquot was pre-incubated with PRV antiserum while the second aliquot was pre-incubated with a non-related serum of swine origin previously tested negative on VNT and ELISA test. Following pre-incubation at 37 °C for 1 h, the mixtures were inoculated into cell cultures. The virus isolate was considered to be ADV when the CPE was inhibited by the antiserum.

### Virus neutralisation test (VNT)

Before the performance of virus neutralisation test the serum samples were heat-inactivated at 56 °C for 30 minutes. PK15 (BS CL 72, IZS, Brescia, Italy) cells used for VNT were maintained in the minimum essential medium (MEM; Gibco BRL, USA) containing 5 % fetal bovine serum (Gibco BRL, USA), with the addition of antibiotics and antimycotics. The ADV used for the VNT was NIA3 strain (CVI, Wageningen, Lelystad, The Netherlands). The VNT was carried out in 96-well microplates. Each serum sample was serially diluted by two folds and each dilution mixed with 100 TCID_50_/50 μL of the virus. After 60 min of incubation at 37 °C, 100 μL of PK15 cells suspension was added to each well. Positive and negative control sera were included and tested the same way. VNT titre was determined after the plates had been incubated for 72 h at 37 °C. The antibody titres of serum samples were expressed as the reciprocal of the highest serum dilution completely inhibiting CPE in the wells. Serum samples neutralising the virus at any dilution were considered to be positive.

### Statistical analysis

Ninety-five per cent confidence intervals (CI) for the standard errors (SE) were estimated from the expression se95% CI = 1 · 96(p [1–p])/n1/2.

To test for significant differences in seroprevalence and also virus-prevalence between gender and age groups, Z-test for two proportions was used. The level of significance was established at 5 %. Statistical analysis was performed using Social Science Statistics website^2^.

## Results

Wild boars were divided by age, determined by hunters, into three groups depending on number of permanent molars according to SCHEDA Ecological Associates, inc. (one molar 6–18 months, two molars 1.5-2.5 year, three molars over 2.5 years of age). The age group 6–18 months old wild boar consisted of 28 animals (females, *n =* 13; males, *n =* 15), the group 1.5-2.5 years old of five wild boars (females, *n =* 3; males, *n =* 2) and the group of animals older than 2.5 years of seven wild boars (females, *n =* 3; males, *n =* 4).

**Fig. 1:**
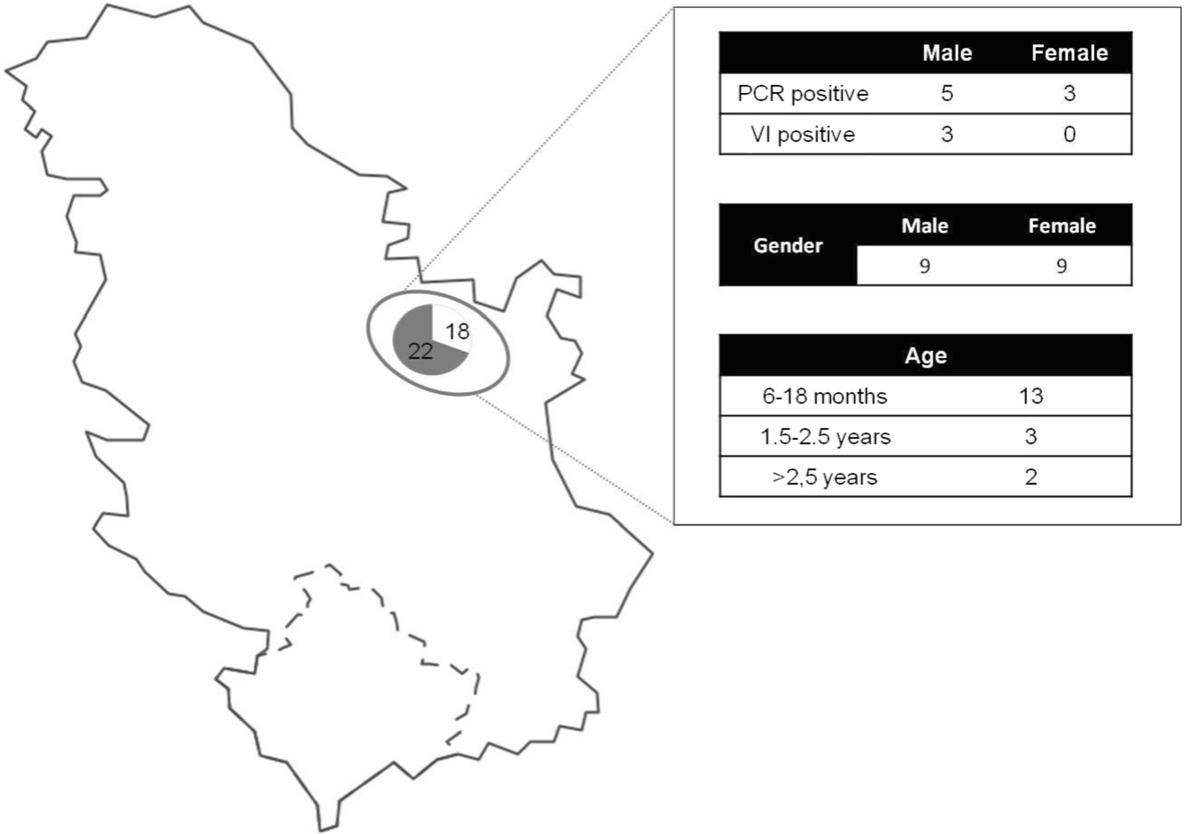
Summarized results of ADV investigations in wild boar from Eastern Serbia where 18 ill wild boars were hunted

The majority of ill animals belonged to the age group 6–18 months old wild boar (females, *n =* 6; males, *n =* 7). Three wild boars belonged to the group 1.5-2.5 years old animals (females, *n =* 1; males, *n =* 2) and two to older than 2.5 years group (females, *n =* 1; males, *n =* 1). The most common symptoms described by hunters for the 18 ill wild boars were depression, dyspnea, slow movement, and no fear of humans. There was no evidence of any previous vaccination. Among 18 ill wild boars, there were 9 males (6–18 months old, *n =* 7; 1.5-2.5 years old, *n =* 1; older than 2.5 years, *n =* 1) and 9 females (6–18 months old, *n =* 6; 1.5-2.5 years old *n =* 2; older than 2.5 years, *n =* 1) (Fig. 1).

**Fig. 2:**
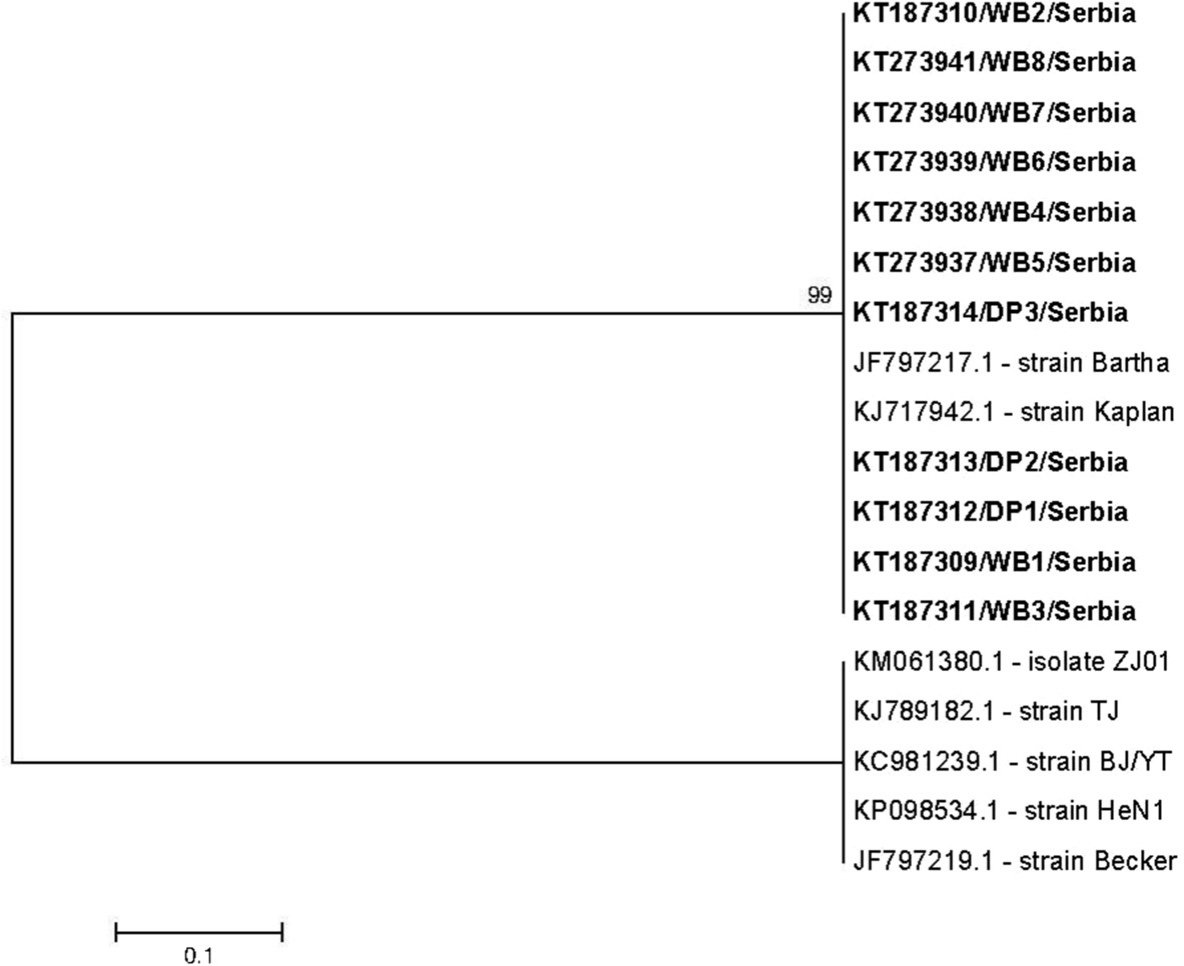
Phylogenetic tree based on us4 sequence constructed using Maximum Parsimony Test. The analysis involved 18 nucleotide sequences. Codon positions included were 1st + 2nd + 3rd + Noncoding. All positions containing gaps and missing data were eliminated. Evolutionary analyses were conducted in MEGA6. Serbian sequences are in bold typewriting, identified by GenBank accession number/species/country. Numbers along the branches represent percentages of 1000 bootstrap iterations. WB: wild boar. DP1: domestic pig, DP2: dog, DP3: domestic pig

**Fig. 3:**
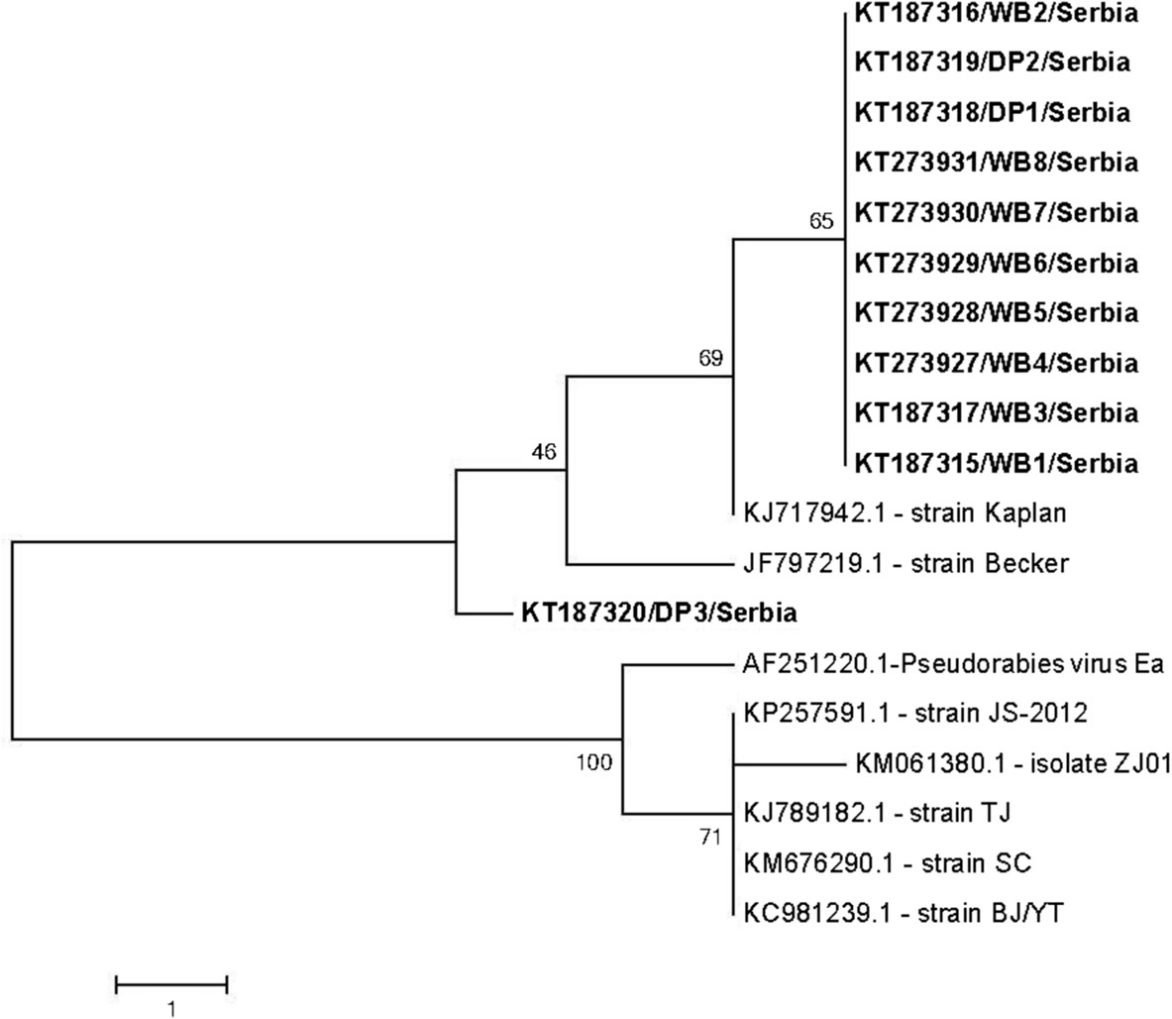
Phylogenetic tree based on us9 sequence constructed using Maximum Parsimony Test. The analysis involved 19 nucleotide sequences. Codon positions included were 1st + 2nd + 3rd + Noncoding. All positions containing gaps and missing data were eliminated. Evolutionary analyses were conducted in MEGA6. Serbian sequences are in bold typewriting, identified by GenBank accession number/species/country. Numbers along the branches represent percentages of 1000 bootstrap iterations. WB: wild boar. DP1: domestic pig, DP2: dog, DP3: domestic pig

**Fig. 4:**
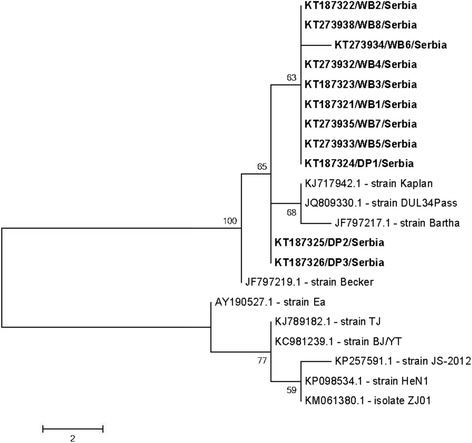
Phylogenetic tree based on ul49.5 sequence constructed using Maximum Parsimony Test. The analysis involved 21 nucleotide sequences. Codon positions included were 1st + 2nd + 3rd + Noncoding. All positions containing gaps and missing data were eliminated. Evolutionary analyses were conducted in MEGA6. Serbian sequences are in bold typewriting, identified by GenBank accession number/species/country. Numbers along the branches represent percentages of 1000 bootstrap iterations. WB: wild boar. DP1: domestic pig, DP2: dog, DP3: domestic pig

AD virus genome was detected in 8 of 40 samples (20 %). All PCR positive (*n =* 8) wild boars were reported to be ill and belonged to the group 6–18 months old wild boar (females, *n =* 3; males, *n =* 5) (Table 1). Based on partial us4, us9 and ul49.5 sequences, which are submitted to and available from NCBI GeneBank under accession numbers: KT187309, KT187310, KT187311, KT187315, KT187316, KT187317, KT187321, KT187322, KT187323, KT273927, KT273928, KT273929, KT273930, KT273931, KT273932, KT273933, KT273934, KT273935, KT273936, KT273937, KT273938, KT273939, KT273940, KT273941, and other available sequences for the same regions, wild boar and domestic animals ADV strains showed high similarity (Figs. 2, 3 and 4). The greatest distance between isolates from wild and domestic animals was observed for us9 gene (3 %), followed by ul49.5 (1 %). There was no distance between those groups of isolates for us4 gene. Comparing isolates from Serbian wild boars and other sequences used for phylogenetic analysis, 32 % distance was determined for us 9gene, 26 % for ul49.5 and 4 % for us4 gene. Certain distance, but highest for ul49.5 gene (6 %), was obvious between wild boar isolates and Bartha vaccine strain. However, no distance was observed between wild boar isolates based on any of three examined sequences.

**Table 1:** Results of serological and molecular tests on spleen and renal samples to demonstrate AD in wild boar *(Sus Scrofa)* in Serbia by detecting anti-ADV antibodies and viral DNA

	Serology results										PCR results			
			Total		VN titre								Total	
Age group	Female	Male	No (%)	95 % Cl	1:8	1:16	1:64	1:128	1:256	1:512	Female	Male	No (%)	95 % Cl
	No (%)	No (%)			No (%)	No (%)	No (%)	No (%)	No (%)	No (%)	No (%)	No (%)		
6 - 18 months	7/13 (53,8)	7/15 (46,7)	14/28(50)	31,48-68,52	5/14(35,7)	3/14(21,4)	3/14(21,4)	3/14(21,4)	0/14(0)	0/14(0)	3/13(23,1)	5/15(33,3)	8/28(28,6)	11,86-45,34
1.5 - 2.5 years	2/3 (66,7)	2/2 (100)	4/5 (80)	44,94-115,06	0/4(0)	0/4(0)	1/4(25)	1/4(25)	1/4(25)	1/4(25)	0/3 (0)	0/3 (0)	0/6 (0)	0
>2,5 years	2/3 (66,7)	3/4 (75)	5/7(71,4)	37,92-104,88	0/5(0)	1/5(20)	2/5(40)	0/5(0)	2/5(40)	0/5(0)	0/3 (0)	0/3 (0)	0/6 (0)	0
Total	11/19 (57,9)	12/21 (57,1)	23/40(57,5)	42,18-72,82	5/23 (21,7)	4/23(17,4)	6/23(26,1)	4/23(17,4)	3/23(13)	1/23(4,3)	3/19 (15,8)	5/21(23,8)	8/40 (20)	7,6-32,4

ADV was isolated on PK 15 cells from 3 of 8 PCR positive samples. All isolates originated from pool of organs (kidney and spleen) from young (6–18 months old) male wild boars which were serologically negative. CPE was evident after 24 hours of incubation for one sample, and after 72 hours for the other two samples. Observed syncytia, different in size, were well developed. In the neutralisation test, all isolates were inhibited with antiserum, confirming the recovered viruses were ADV. No further virus isolations were obtained from negative samples by further blind passage.

Twenty-three out of 40 (57.5 %) serum samples had neutralising antibodies in the VNT against ADV.

Eight ill wild boars were negative by VNT, out of which four were PCR positive.

**Fig. 5:**
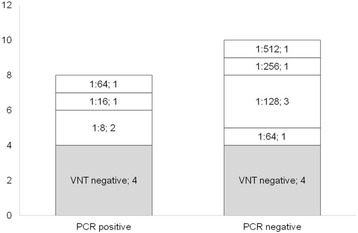
VNT results of PCR positive wild boars

The other four PCR positive animals were seropositive by VNT, two at a titre of 1:8, and one each at titres of 1:16 and 1:64 (Fig. 5). The rest of ill animals had titres 1:64 (*n =* 1), 1:128 (*n =* 3), 1:256 (*n =* 1) and 1:512 (*n =* 1). Out of 9 ill males, 5 were negative by VNT, two had titre 1:8, and one each 1:64 and 1:256. Out of 9 ill females, 3 were negative by VNT and 6 had certain VN titre (1:16, *n =* 1; 1:64, *n =* 1; 1:128, *n =* 3; 1:512, *n =* 1)

Thirteen of 22 healthy wild boars were seropositive (Table 1), three at titre 1:8 (6–18 months old, *n =* 3), three at titre 1:16 (6–18 months old, *n =* 2; older than 2.5 years, *n =* 1), four at titre 1:64 (6–18 months old, *n =* 2, 1.5-2.5 year old, *n =* 1; older than 2.5 years, *n =* 1).

However, the results of this study did not show any statistically significant difference between neither genders nor age categories (*P* > 0.05)

PRRSV, PCV2, SIV, PRCV and PPV were not detected by molecular methods in samples from the 18 ill wild boars. Anti-PPV2 antibodies were detected in 11 of 18 serum samples (61.1 %). No antibodies to other viruses were detected.

No PRRSV, PCV2, SIV, PRCV, PPV and ADV were detected by molecular tests on samples from the 22 clinically healthy wild boars. Seroprevalence for ADV and PPV in that group were 59.1 % (*n =* 13/22) and 63.6 % (*n =* 14/22) respectively. No antibodies to other viruses were detected.

## Discussion

Previous serological surveys conducted in wild boar in Serbia were limited but suggested a relatively high AD seroprevalence [28]. Lazic et al. (2014) [28] reported the mean prevalence of 38.21 % with the highest percentage among population older than 2.5 years - 46.86 %. However, neither virus nor clinical disease were reported in wild boar in Serbia^3^.

Nasal swabs, oropharyngeal fluids and tonsil biopsies were reported as the samples of choice to be collected from live animals, and brain, tonsils [29], spleen and lung^4^ from dead animals. Also, virus could be successfully isolated from vaginal secretions, ejaculates, milk, urine, rectal swab, even before the onset of clinical symptoms [20]. In this study, ADV was isolated from spleen and renal tissue samples, reported to be virus positive during active infection [29]. Wittnamm et al. [30] showed that, after experimental infection in piglets, the virus could not be detected in spleen, liver, and mediastinal lymph nodes; but could be demonstrated in kidneys, CNS and peripheral lymph nodes. Since we used samples already submitted for Classical Swine Fever monitoring, other tissue samples were not available. In this study, ADV was isolated on cell culture from 3 out of 8 PCR positive samples. Those 3 samples originated from seronegative wild boars. ADV was not isolated from one more seronegative but PCR positive animal. The discrepancy between results of virus isolation and PCR occurred due to the characteristics of the applied tests, their limitations and advantages. Presence of antigen-antibody complexes, cytotoxic substances, storage and transport conditions could significantly affect virus isolation requiring viable viruses in sample, in contrast with molecular tests detecting only genome fragments. Bearing in mind the characteristics of the applied tests, we presume that virus isolation failed in seropositive animals due to presence of antibodies, and in one sample from seronegative animal due to dependence of virus isolation on sample quality. The isolation of ADV from spleen and renal tissues and low serum antibody titres indicated those were cases of active ADV infection. Virus isolation was performed on the PK15 cell line where the virus induced syncytia, previously associated with virus pathogenicity [25]. Bitsch et al. [25] concluded that such isolates were highly pathogenic for pigs and cattle, although conversely lack of syncytia formation was not considered as an indicator of attenuation. The highly developed syncytia observed in the study supported the conclusion the cause of the depression and other symptoms reported by the hunters was active AD.

The herpesvirus genome is highly conserved and most genes are not sufficiently variable for high–resolution phylogenetic analysis. With ADV slow rate of genetic evolution, genetic diversity within a population may be primarily caused by a high prevalence of infection [27].

According to partial ul44 gene, 5 groups of ADV could be distinguished, A-E. Strains isolated from feral pigs belonged to groups A and C, strains isolated from domestic pigs belonged to B and D groups. Group E was formed mainly of strains from Eastern regions, primarily China [4]. Fonseca et al. (2014) [26] found, analysing other genes, two clusters forming which separated viral isolates from East and West.

In this study, three partial sequences, (us4, us9 and ul49.5) were analized in order to increase the probability of detecting variation between the isolates from wild and domestic animals. However, the overall distance between isolates (eight wild boars, three domestic animals) as well as between viruses detected in the wild and domestic animals was negligible. This finding was consequence of the insufficient number of samples and a short sampling period, along with limited number of available sequences for those three regions in GeneBank. The biggest differences were observed for the us9 gene which encoded 11 kDa protein responsible for axonal transport, followed by ul49.5 gene encoding gN, involved in viral morphogenesis and membrane fusion. Analysis of gG sequence from Serbian wild boar and domestic animals isolates showed that the gene was highest conserved among examined genes. However, certain distance between isolates from wild and domestic animals indicated different origin of those strains, but no difference between isolates from wild boar, based on three genome sequences. The low genetic diversity, which is characteristic of herpesviruses, is also responsible for the low bootstrap values observed in the constructed trees.

Similar findings were reported by Keros et al., [31]. The authors reported that genetically identical viral strains belonging to Clade A were circulating in Croatia in wild boar and domestic pigs, according to the gC sequences derived from six domestic pigs and one wild boar. Furthermore, sequencing the gC genome fragment of ADV isolated from two hunting dogs confirmed the presence of Clade A genotype, similar to strains derived previously from domestic pigs and wild boars in Croatia [32].

Experimental infections showed that wild boar ADV isolates could be species adapted, leading to a different clinical and immunological response than in domestic pigs [1].

Manifested clinical symptoms are a product of virus virulence and dose, age of host animal and its reproductive and immunological status. They are the most observed in piglets and sows. The most affected age category in our study was young wild boars, 6–18 months old. Clinical signs expected in that category caused by ADV were rather nonspecific, i.e. depression, appetite loss, fever, cough etc. Pathomorphological signs were not typical or were absent. Such observations were reported by the hunters, but could not be linked to any specific disease.

Numerous reports have suggested that females have been more frequently infected with ADV [33–35]. Females are considered to be at higher risk of infection with ADV due to their different behaviour. Females live in groups in which contacts between animals are more frequent than among solitary males. Additionally, older and socially more active adults are at a higher risk of infection [36]. However, the results of this study did not show that there was any statistically significant difference between genders or age categories (*P* > 0.05). However, all ADV positive animals in this study were 6–18 months of age and it was consistent with the results from the outbreak in Spain where most affected animals were 4–8 months old [11]. Sample bias could not be excluded due to the majority of samples (70 %) being acquired from that age class.

Serological results showed that 50 % of wild boars 6–18 months of age were ADV antibody positive. High VN titres, higher than 1:128, which were seen in the age groups 1.5-2.5 years and more than 2.5 years, might be due to the higher stimulation of the immune system by a putative reactivated latency. Low VN titres in young animals and no neutralising antibodies in ADV positive animals indicated that this was a recent infection rather than latent virus reactivation. Verin et al. [37] demonstrated that serology tests could be negative in recently infected animals. Up to 45 % of animals with the virus in tonsils could be seronegative [34].

All PCR positive animals were hunted in one hunting ground (area of 44349 ha) with a total number of wild boars estimated at 100, leading to an estimated morbidity of 18 %. The seroprevalence in that hunting ground was 57.5 %, and in a previous survey 57 % (unpublished data). The seroprevalence in Spain affected by an outbreak in wild boar was also above 50 % [11].

ADV transmission depends on a direct contact between animals [20] but since the outbreak occurred in the less-inhabited area of Serbia, there was a low probability of contact between wild and domestic pigs, and consequently a low risk of ADV transmission to domestic pigs. So although feral pigs and wild boars were recognised as reservoirs of ADV [13, 38], the detection of active ADV infection within wild boar did not indicate they were a likely source of infection for domestic pigs. Nonetheless, the findings were supported that wild boar species was able to maintain infection without the presence of domestic pigs [35] and with no discernible effect on the population structure [37].

ADV infection affects the reproduction and health status of wild boars, particularly in piglets and the higher presence of viral genome in piglets and genital swabs also indicating both vertical and venereal transmission [37].

Nevertheless, a relatively low risk of transmission to domestic pigs still exists since there is evidence that ADV was spontaneously shed in the nasal secretion of a sow after parturition 19 months post infection with no clinical signs [39]. Additionally, daily migrations and frequent contacts with different subjects plays significant role in virus spreading [35].

There are many reports suggesting increase of population of wild boar in Europe, causing significant damage to agriculture. According to hunting associations, wild boar population in Serbia is stable. But domestic pigs production, still based on back yard system, with low or no biosecurity measures applied, is the critical place where the virus can enter domestic pig population and further on into intensive production systems.

The accurate diagnosis of AD can also be affected by previous vaccination of domestic pigs. There is evidence that the viruses from modified live vaccine (MLV) can be shed from the oronasal cavity [40], but Terpstra and Pol [41] showed that vaccination with MLV vaccines was not likely to interfere with the laboratory diagnosis of ADV field strains. However, since the attenuated virus replicates in the body and can be excreted, transmission to other animals, reverse mutations and genetic recombination with field strains cannot be excluded [42]. The most commonly used AD vaccine in Serbia for domestic pig vaccination is made using the attenuated Bartha strain, which minimizes field virus excretion comparing to inactivated vaccines [43]. Though, Gielkens at al*.* [44] reported two cases where the ADV field isolate, recovered from diseased pigs, was suspected to be of attenuated vaccine virus origin, they also did not exclude the possibility that the disease was caused by a virulent virus present in the herd before vaccination. Due to the long term vaccination of wild boars in Serbia, it was interesting to compare genome sequences between wild boar isolates and Bartha strain. Greatest distance of 6 % has been shown for ul49.5 gene, where Bartha strain, along with Kaplan and DUL34pass strain, forms separated cluster.

## Conclusions

Successful isolation of AD virus from tissues of wild boars showing signs of illness (depression, dyspnoea, slow movement) indicates that an AD outbreak did occur in Eastern Serbia during the winter 2014–2015. The hypothesis that this was a recent infection is supported by serological results showing low VN titre or no titre in virus positive animals. Furthermore, other viral diseases that could affect the health status of wild boar were excluded.

Genetic analysis based on three partial sequences showed that isolates from both domestic pigs and wild boars from East Serbia were very close to each other. Nevertheless, we presume that due to the different status and management between wild boar and domestic pigs, ADV can evolve but to follow this process genes, that exhibit evidence of positive selection, should be selected.

## Abbreviations

AD, Aujeszky’s disease; ADV, Aujeszky’s disease virus; CI, confidence intervals; CPE, cytopatic effect; CSF, Classical Swine Fever; DNA, deoxyribonucleic acid; ELISA, enzyme-linked immunosorbent assay; NCBI, National Centre for Biotechnology Information; OIE, World Organisation for Animal Health; PCR, Polymerase Chain Reaction ; PCV2*, Porcine Circovirus2* ; PPV, *Porcine Parvovirus*; PR, Pseudorabies; PRCV, *Porcine Respiratory Corona virus*; PRRSV, *Porcine reproductive and respiratory syndrome virus*; PRV, Pseudorabies virus; SE, standard error; SHV1, *Suid Herpesvirus 1*; SIV, *Swine Influenza Virus*; TCID, tissue culture infective dose; UK, United Kingdom; VNT, virus neutralisation test; WAHID, World Animal Health Information System

## Footnotes

1 See: Official Journal of the European Union: http://eur-lex.europa.eu/legal-content/EN/TXT/PDF/?uri=CELEX:32008D0185&from=EN (accessed March 2015)

2 See: http://www.socscistatistics.com/pvalues/normaldistribution.aspx (accessed March 2015)

3 See: World Animal Health Information Database (WAHID): http://www.oie.int/wahis_2/public/wahid.php/Diseaseinformation/Immsummary (accessed March 2015)

4 See: The Merck Veterinary Manual: http://www.merckvetmanual.com/mvm/nervous_system/pseudorabies/overview_of_pseudorabies.html (accessed March 2015)
